# Leveraging genomics, transcriptomics and epigenomics to understand chemoimmunotherapy resistance in chronic lymphocytic leukemia

**DOI:** 10.20517/cdr.2023.98

**Published:** 2024-02-28

**Authors:** Shin Yeu Ong, Lili Wang

**Affiliations:** ^1^Department of Hematology/Hematopoietic Cell Transplantation, City of Hope National Medical Center, Duarte, CA 91010, USA.; ^2^Department of Hematology, Singapore General Hospital, Singapore 169608, Singapore.; ^3^Department of Systems Biology, Beckman Research Institute, City of Hope National Medical Center, Duarte, CA 91010, USA.

**Keywords:** Chronic lymphocytic leukemia, chemoimmunotherapy, resistance

## Abstract

Patients with chronic lymphocytic leukemia (CLL) have differing clinical outcomes. Recent advances integrating multi-omic data have uncovered molecular subtypes in CLL with different prognostic implications and may allow better prediction of therapy response. While finite-duration chemoimmunotherapy (CIT) has enabled deep responses and prolonged duration of responses in the past, the advent of novel targeted therapy for the treatment of CLL has dramatically changed the therapeutic landscape. In this review, we discuss the latest genomic, transcriptomic, and epigenetic alterations regarded as major drivers of resistance to CIT in CLL. Further advances in genomic medicine will allow for better prediction of response to therapy and provide the basis for rational selection of therapy for long-term remissions with minimal toxicity.

## INTRODUCTION

Recent large-scale whole-genome sequencing and multi-omics studies have revealed marked heterogeneity in chronic lymphocytic leukemia (CLL) and identified novel genetic and epigenetic alterations causing resistance to chemoimmunotherapy (CIT) in CLL, leading to a better understanding of the pathogenesis of CLL. While historical CIT combinations have allowed patients with CLL to achieve good responses formerly, targeted therapies (e.g., bruton tyrosine kinase and B-cell lymphoma-2 inhibitors) have impressive activity in high-risk genomic subgroups and have largely replaced the role of CIT for all patients with CLL in certain countries. Despite this, there may be a select group of patients aged less than 65 years old, with immunoglobulin heavy-chain variable region (*IGHV)-*mutated disease and without high-risk cytogenetic abnormalities including del(11q) and del(17p), in whom fludarabine, cyclophosphamide, and rituximab (FCR) can offer durable remissions, especially if financial toxicity is a concern^[1]^.

While the role of CIT continues to diminish in frontline CLL, long-term follow-up of patients with mutated *IGHV* and without tumor protein p53 (*TP53*) aberrancy treated with FCR in a phase II clinical trial showed sustained progression-free survival (PFS) of 54% at 12 years, with no relapses observed beyond 10.4 years follow-up^[2]^, suggesting FCR could cure a significant portion of patients with good risk disease. The CLL8 trial, which evaluated fludarabine/cyclophosphamide (FC) against FCR, also showed, at a median 5.9 years of follow-up, that some patients without del(17p) remained in long-term remission^[3]^. Lastly, the GAIA-CLL13 trial randomized young and fit patients with CLL without *TP53* aberrations to receive FCR/bendamustine-rituximab, time-limited venetoclax-obinutuzumab, or ibrutinib with venetoclax-obinutuzumab, and found no PFS benefit among patients with mutated *IGHV*^[4]^. When selecting FCR as the treatment for frontline CLL, it is critical to carefully counsel the patient about potential toxicities, including cytopenias with concomitant infectious complications, and a small but increased risk (6.3%) of secondary acute myeloid leukemia/myelodysplastic syndrome^[3]^. In this review, we will detail the genomic, transcriptomic, and epigenetic alterations that portend poor outcomes in patients with CLL treated with CIT. These emerging mechanisms of resistance to CIT can guide clinical care and assist in the selection of the most appropriate therapy for patients with CLL.

## GENOMIC ALTERATIONS AND CHEMOIMMUNOTHERAPY RESISTANCE

Common genomic alterations in CLL that are associated with chemotherapy resistance are detailed below and summarized in Table 1.

**Table 1:** Recurrently mutated genes in CLL and mechanisms of chemoresistance

**Biomarker**	**Mechanisms of resistance**	**Prevalence before treatment and at progression**	**Implications**	**Ref.**
Del(17p)/*TP53* mutations	Genomic instability, survival advantage, and reduced DNA damage response	5% to ~30%	Poor response to CIT	[5-8]
Unmutated *IGHV* gene	Increased B-cell receptor signaling capacity	~40% to 70%-80%	Poor response to CIT	[3,46]
*BIRC3* mutations	Upregulation of non-canonical NF-κB signaling pathway	2%-6% to 8%	Poor response to CIT	[19-21]
*NOTCH1* mutations	Transcriptional activation of cell survival and proliferation and reduced expression of CD20	8%-10% to 30%	Poor response to CIT and anti-CD20 mAbs	[24-26]
*ATM* mutations	Activates cell cycle checkpoints to induce apoptosis of cells with excess DNA damage	20% to 30%	Poor response to CIT	[14]
*POT1* mutations	Increased chromosomal aberrations and genomic instability	5%-8% to 13%	Poor response to CIT, reduced overall survival	[16,17]
*FBXW7* mutations	Interferes with *NOTCH1* signaling by degrading *NOTCH1* intracellular domain by	2% to 6% in newly diagnosed	Poor response to anti-CD20 antibody	[29,30]
*SF3B1* mutations	Impaired DNA damage response and increased NOTCH signaling	3% to 17%	Poor response to CIT	[33-35]
*XPO1* mutations	Mislocalization of tumor suppressor proteins	Frequency similar to naïve and relapsed	Poor response to CIT	[37-39]
*RPS15* mutations	Increases *TP53* degradation causing global changes towards a hyperproliferative cellular state	5% to 20%	Poor response to CIT	[40-43]
*MGA* mutations	Shifts balance towards *MYC*-dependent activation of cell proliferation and transformation	3% to 16%	Poor response to CIT	[31,32]

CLL: Chronic lymphocytic leukemia; CIT: chemoimmunotherapy; IGHV: immunoglobulin heavy-chain variable region; *BIRC3*: baculoviral IAP repeat-containing protein 3; NF-κB: nuclear factor-κB; *NOTCH1*: NOTCH receptor 1; *ATM*: ataxia telangiectasia-mutated; POT1: protection of telomeres 1; *FBXW7*: F-box and WD40 repeat domain containing-7; CD20: cluster of differentiation 20; *XPO1*: exportin 1; RPS15: ribosomal protein S15; *TP53*: tumor protein p53; MGA: max gene associated; *MYC*: myelocytomatosis oncogene.

### DNA damage response and cell cycle control

#### TP53

*TP53* mutation and/or deletion (*TP53* aberrancy) is the most important consideration when deciding about treatment using chemotherapy, since many studies, including prospective clinical trials, have shown poorer responses and shorter survival after chemotherapy treatment in patients with *TP53* mutations or deletions of 17p^[5-8]^. *TP53* encodes p53, a key mediator of the DNA damage response pathway that controls cell cycle arrest and apoptosis. Chemotherapy induces DNA damage, which, if not repaired by physiological DNA repairing enzymes, leads to a series of intracellular signaling related to *TP53*, causing cell apoptosis or preventing cell proliferation. *TP53* aberrancies, by preventing apoptosis, result in the propagation of cells with an increasing number and type of chromosomal and genetic abnormalities. Targeted therapies attack specific genetic biomarkers independently of the p53 pathway and should thus be chosen in patients with known *TP53* aberrancies. Deletions of chromosome 17p can be detected via fluorescence in situ hybridization (FISH), and an additional 5% of patients with CLL carry *TP53* mutations without concomitant del(17p)^[5,8]^. The European Research Infrastructure Consortium (ERIC) guidelines suggest using Sanger sequencing (10%-20% sensitivity) or next-generation sequencing (NGS) to detect these *TP53* mutations^[9]^. While the significance of low-burden *TP53* mutations with < 10% VAF that are detected using NGS remains to be established^[10]^, some studies have shown that chemoimmunotherapy leads to clonal expansion of small *TP53* subclones, leading to a chemo-refractory phenotype with unfavorable prognosis at relapse^[11]^. In contrast, novel agents did not lead to the propagation of adverse subclones. In addition, CLL with *TP53* abnormalities are associated with higher genomic complexity than CLL without, and this may have additional adverse prognostic implications for poor outcomes such as unmutated IGHV status, del(17p) or del(11q)^[12]^.

#### Ataxia telangiectasia-mutated

The ataxia telangiectasia-mutated (*ATM*) gene maps to chromosome 11q22-q23 and *ATM* mutations are usually present at diagnosis in 20% of cases^[13]^. The *ATM* is a protein kinase that, following induction of DNA double-strand breaks, synchronizes DNA repair, activation of cell cycle checkpoints, and induction of apoptosis with elimination of cells with excessive DNA damage. In patients enrolled in the CLL4 trial treated with first-line fludarabine and cyclophosphamide, *ATM* mutation was associated with reduced overall and progression-free survival^[14]^. The residual *ATM* allele was mutated in 36% of cases of patients with 11q deletions, and biallelic *ATM* disruption led to complete loss of *ATM* function with the worst outcomes to CIT^[15]^.

#### Protection of telomeres 1

The protection of telomeres 1 (*POT1*) encodes a protein member of the shelterin complex, which regulates telomere integrity. The shelterin complex compacts telomeric chromatin, which protects chromosome ends from instability, abnormal chromosome segregation, and inadequate recombination. Somatic mutations of the *POT1* gene occur in 5%-8% of patients with CLL, affecting the ability of the protein to bind to telomeric DNA, leading to uncapping of telomere ends and chromosomal aberrations^[16,17]^. In the CLL11 trial, patients were randomized to receive chlorambucil, chlorambucil-rituximab, or chlorambucil-obinutuzumab. Patients with *POT1* mutations receiving CIT had shorter overall survival (OS) independent of variables including Binet stage, elevated β2-microglobulin, unmutated *IGHV* status, and complex karyotype^[16]^.

### Nuclear factor-κB pathway

#### Baculoviral IAP repeat-containing protein 3

Baculoviral IAP repeat-containing protein 3 (*BIRC3*) is a negative regulator of non-canonical nuclear factor-κB (NF-κB) signaling, and inactivation or deletion of *BIRC3* in CLL results in constitutive NF-κB pathway activation, which provides pro-survival signals to the leukemic clone through upregulation of several anti-apoptotic genes^[18]^. *BIRC3* frameshift insertion/deletions or nonsense mutations are detected in around 4% of CLL patients at diagnosis, and since it is located on chromosome 11 in proximity to *ATM,* 11q deletions include the *BIRC3* locus 83% of the time^[14]^. *BIRC3* alterations are also associated with unmutated *IGHV* gene status and represent significant prognostic factors for unfavorable PFS after FCR and Obinutuzumab-chlorambucil^[19-21]^. Patients with *BIRC3* mutations tended to have shorter overall survival compared with the wild-type group after treatment with CIT^[22]^.

### NOTCH signaling

#### NOTCH receptor 1

NOTCH receptor 1 (*NOTCH1*) codes for a transmembrane receptor that, upon binding to its ligand, is cleaved to generate transcriptionally active nuclear intracellular domain of *NOTCH1*, which transactivates genes involved in cell survival and proliferation, including myelocytomatosis oncogene (*MYC*) and components of the NF-κB pathway^[23]^. *NOTCH1* mutations are present in 8%-12% of untreated patients, which can increase to 13%-21% in patients who are refractory to CIT^[24]^*. NOTCH1* mutations are also enriched in patients with trisomy 12 and subset #1 and #8, which are associated with poor prognosis^[25,26]^. *NOTCH1* mutated patients may not benefit from the addition of rituximab to FC due to lower cluster of differentiation 20 (CD20) expression leading to a lower extent of complement-dependent cytotoxicity induced by type I anti-CD20 monoclonal antibodies^[24]^. In CLL8, patients with mutated *NOTCH1* treated with FC *vs*. FCR had a similar 5-year PFS rate of 25.8% *vs*. 26.7%^[7]^. Similarly, in a phase III trial evaluating chlorambucil alone against chlorambucil and ofatumumab, *NOTCH1* mutations predicted refractoriness to ofatumumab^[21]^. However, obinutuzumab, a type 2 anti-CD20 monoclonal antibody with enhanced antibody-dependent cell-mediated cytotoxicity and increased direct cell-mediated cytotoxicity may retain efficacy in *NOTCH1* mutated cases^[27]^. In the CLL11 trial, chlorambucil with obinutuzumab led to better progression-free and overall survival than chlorambucil with rituximab independent of *NOTCH1* genetic lesions^[28]^.

#### F-box and WD40 repeat domain containing-7 mutations

In CLL, F-box and WD40 repeat domain containing-7 (*FBXW7*) mutations occur at a frequency of 2% to 6% and commonly affect the WD40 domain of an E3 ubiquitin ligase that interacts with proteins that are subsequently subject to proteasomal degradation. Mutations of *FBXW7* resulted in an increase of activated *NOTCH1* intracellular domain (NICD) by preventing its degradation by proteasomes^[29]^. Thus, NICD accumulates in the nucleus thereby sustaining *NOTCH1* target gene expression, leading to dysregulated *NOTCH1* signaling without mutations. Among CLL patients who harbor *NOTCH1* mutations, treatment with rituximab does not result in the expected increase in PFS compared with treatment with chemotherapy regimens that do not contain rituximab, and *FBXW7* serves as another biomarker of CD20 monoclonal antibody resistance^[30]^.

### *MYC* deregulation

#### Max gene associated

Max gene associated (*MGA*) is a functional *MYC* suppressor that was found to be mutated in 3% of newly diagnosed CLL, 16% of fludarabine-refractory CLL, and 36% in Richter’s transformation^[31]^. MGA is a protein that forms a heterodimer with MAX and this heterodimer antagonizes *MYC*-dependent activation of transcription of target genes that drive cell proliferation, growth, and cellular transformation^[32]^. Genetic lesions including focal and recurrent gene deletions or truncating point mutations targeting *MGA* are enriched in high-risk CLL with chemoresistance and increase the tendency of transformation to an aggressive phenotype^[32]^.

### RNA and ribosomal processing pathway

#### SF3B1

*SF3B1* encodes a component of the spliceosome, which is involved in the RNA splicing of precursor messenger RNA and the removal of introns in protein-encoding genes^[33]^. The *SF3B1* gene is mutated in 3.6%-9.3% of patients with newly diagnosed CLL, which increases to 17% at relapse, and is enriched in patients with subset 2 disease^[34]^. Transcriptomic profiling revealed that multiple pathways are altered by *SF3B1* mutations, including DNA damage response, cell cycle response, and NOTCH signaling^[35]^. *SF3B1* mutations have been associated with fludarabine resistance and poor outcomes with shorter PFS in CLL8^[33]^. Moreover, co-expression of *SF3B1*-K700E with *ATM* deletion in murine B cells led to the onset of CLL, confirming the causative effects of this mutation in the onset of CLL^[36]^.

#### Exportin 1

The exportin 1 (*XPO1*) gene is located on chromosome 2 and encodes a protein that mediates the translocation of numerous RNA and cellular regulatory proteins from the nucleus to the cytoplasm. The frequency of *XPO1* mutations was similar in treatment-naïve and relapsed patients (~7.5%-10%); mutations lead to cytoplasmic mis-localization of tumor suppressor proteins (*TP53*), cell cycle inhibitors, and growth receptor proteins (e.g., STAT3)^[37]^. *XPO1* mutations are associated with resistance to FCR and decreased PFS after chemoimmunotherapy, independent of *IGHV* mutation status or Binet stage^[38]^. Expression of *Xpo1*-E571K mutation in murine B cells resulted in the onset of CLL as early as 7 months of age^[39]^. Molecular characterization of these CLL cells revealed that mutant *XPO1* altered the nucleo-cytoplasmic distribution of hundreds of proteins in a sequence-specific manner that promoted oncogenesis, rendering the sensitivity of these cells to inhibitors of nuclear export. These results provide a rationale for testing nuclear export inhibitor drugs in cases resistant to FCR.

#### Ribosomal protein S15

Ribosomal protein (RP) *S15* is recurrently mutated in ~5% of untreated CLL, and mutations are associated with a reduced duration of response and resistance to fludarabine-based chemotherapy^[40]^. *RPS15* mutations can affect up to 20% of CLL patients relapsing after FCR, and approximately a third of *RPS15* mutated patients also carry a *TP53* aberrancy^[41]^*.* Wild-type RPS15 binds to mouse double minute 2 homolog (MRM2) and inhibits its E3 ubiquitin ligase activity, abrogating p53 degradation, leading to upregulation of *TP53* target genes, and cell apoptosis. Mutant RPS15 leads to increased *TP53* degradation through ubiquitination and causes global changes of the proteome towards a hyperproliferative cellular state^[42]^. In the CLL8 trial, the mutation was associated with a shorter PFS, but with no significant reduction in OS^[40]^. In agreement with its critical role in driving aggressive type of CLL, co-expression of *RPS15* mutation and *TP53* deletion in murine B cells altered translation and *MYC* activation and led to the onset of B cell malignancy with a shortened latency compared to mice with *RPS15* mutation alone^[43]^.

### MAPK-ERK pathway

#### KRAS and NRAS

The *KRAS* gene encodes a member of the GTPase superfamily and plays an important role in the regulation of cell proliferation. *KRAS* exon 2/3 mutations occur in ~6% of CLL and are associated with trisomy 12 CLL, promoting an increase in RAS/ERK downstream signaling and constitutive activation of the MAPK signaling pathway^[44]^. In the CLL11 trial, the presence of *KRAS* exon 2/3 mutation was associated with refractoriness to chemoimmunotherapy, especially to chlorambucil-rituximab^[16]^. The *NRAS* gene has intrinsic GTPase activity, is located at the 1p13.2 band, and encodes a membrane protein that alternates between the Golgi apparatus and the plasma membrane. The frequency of *NRAS* missense mutation in CLL patients is ~2.4%^[44]^, mostly at subclonal levels. There is a mild association between the *NRAS* mutation and trisomy 12, and co-occurrence of *NRAS* with the *KRAS* mutation is associated with refractoriness to first-line treatment using chlorambucil-rituximab^[16]^.

#### IGHV mutation status

The degree of somatic hypermutation of the immunoglobulin heavy-chain variable region (*IGHV*), which encodes part of the B-cell receptor, identifies two disease subtypes. Somatic hypermutation is a physiological process that generates immunoglobulin diversity during normal B cell maturation and remains stable throughout the course of the disease. *IGHV* is mutated ≥ 2% from a reference germline sequence in patients with mutated *IGHV* (M-CLL), while IGHV sequences are mutated less than 2% from germline sequence in patients with unmutated *IGHV* (U-CLL)^[45]^. U-CLL has increased B-cell receptor signaling capacity and poorer prognosis. Among patients receiving CIT, the *IGHV* mutation status affects the kinetics of relapse and thus PFS, and almost all U-CLL patients are projected to progress after chemoimmunotherapy^[3,46]^. Patients with U-CLL compared with M-CLL had worse outcomes after FCR in CLL8 (5-year PFS 33.1% *vs*. 66.6%), after bendamustine-rituximab in CLL10 (median PFS 33.9 *vs*. 68.9 months), and after chlorambucil-based regimens in CLL11^[47]^.

### Transcriptome

#### Gene expression clusters

While the current classification of CLL still relies largely on genomic alterations, novel insights from nongenomic RNA sequencing data have led to consensus clustering of gene expression subgroups with unique clinical and biological significance. In a recent study by Knisbacher *et al.*, where whole-exome sequencing and whole-genome sequencing data were analyzed from 1,074 CLL cases, the authors defined eight gene expression clusters (EC) with characteristic transcriptomic profiles and differing prognoses^[48]^. U-CLL belonged to two subtypes (EC-u1, EC-u2), while M-CLL belonged to four subtypes (EC-m1, ECm2, EC-m3, and EC-m4). In the remaining two clusters, EC-I was associated with the intermediate CLL epitype, while EC-II was not associated with any previously defined CLL group. The two EC-u clusters had poorer disease outcomes. In particular, the *OSBPL5* gene was found to be upregulated in EC-u1 and was the top expression marker predicting shorter PFS after treatment with FCR in a transcriptional profiling study on samples obtained from 101 treatment-naïve CLL patients at M.D. Anderson Cancer Center^[49]^.

#### MicroRNA

MicroRNAs are short non-coding RNAs that modulate post-transcriptional gene expression and are involved in the regulation of many physiological and pathological processes. Several studies identified microRNA expression profiles in CLL cells that were associated with resistance to fludarabine and rituximab. Both lower expression of miR-34a and high miR-155 expression have been associated with poor response to fludarabine^[50-52]^. miR-34a is a microRNA component of the p53 pathway and directly affects *CDK4*, *CDK6*, *CCND1*, *CCNE2*, and *MET* proto-oncogene^[53]^. A high expression level of miRNA-155 can attenuate histone deacetylase 4 and BCL6 transcription repressor expression, leading to the activation of oncogenes associated with increased cell division and apoptosis inhibition^[54]^.

#### RNA splicing

Dysregulated RNA splicing is a prominent molecular trait found across various tumor types. Splicing alterations in cancer stem from recurrent mutations and changes in the expression of regulatory factors responsible for splicing control. RNA splicing dysregulation can drive tumorigenesis through a multitude of mechanisms, including promoting cell proliferation, inhibiting apoptosis, enhancing migration and metastasis, fostering resistance to chemotherapy, and enabling evasion of the immune system’s surveillance. In CLL, mutations in splicing factors *SF3B1* and *U1snRNA* have been reported previously and are linked to poor prognosis. CLL cells with these mutations all have transcriptome-wide splicing changes^[55,56]^. In addition, recent studies also revealed that RNA splicing dysregulation in CLL cannot be fully explained by genetic alterations of the splicing factor SF3B1 alone but is contributed by upregulation of splicing factors at the protein level compared to normal B cells. This could be caused by post-transcriptional regulation in CLL cells, such as RNA epigenetic modification^[57]^. More importantly, splice variants can be used to stratify CLL patients into indolent and aggressive subgroups^[58]^. It remains elusive how RNA splicing dysregulation is related to FCR resistance and whether RNA splicing inhibitors currently in clinical trials may override the resistance.

### Epigenome

#### DNA methylation change subgroup

Epigenetics comprises modifications of the DNA molecule that affect gene expression through transcription, without changes to the nucleic acid sequence. Epigenetic mechanisms include DNA methylation, histone modifications, chromatin accessibility, and gene regulation through non-coding RNAs. DNA methylation leads to the addition of a methyl group to 5’carbon of cytosine in cytosine guanine dinucleotides (CpG), and is the most extensively studied epigenetic change studied in CLL. CLL has been clustered into three epigenetic subgroups based on DNA methylation profiles, as follows: naïve B-cell like CLL (n-CLL), memory B-cell like CLL (M-CLL), and intermediate group (i-CLL) from antigen-exposed B-cell that has not passed the germinal center^[59-61]^. The majority (97%) of n-CLL have unmutated *IGHV* genes, while most M-CLL have mutated *IGHV*. The i-CLL group has a borderline IGHV mutational load and showed higher (38%) use of *IGLV3-21*^R110^ mutation which promotes autonomous B-cell receptor signaling. While these patients have borderline mutated *IGHV* genes, their phenotype resembles unmutated CLL, with aggressive disease characteristics and adverse clinical outcomes with CIT^[62]^. In addition, a higher cumulative mitoses (epiCMIT) score within individual epitypes, which reflects a more extensive CLL proliferation history, was associated with a worse prognosis^[63]^. Another epigenetic biomarker identified was DNA hypermethylation at *HOXA4*. Both *HOXA4* hypermethylation during CLL progression and relapse and loss of HOXA4 expression were associated with resistance to chemotherapeutic agents including fludarabine^[64]^.

#### RNA epigenetic modification

Increasing evidence emphasizes the crucial functions of N^6^-methyladenosine (m^6^A) modification in mRNA during cancer initiation, progression, and drug resistance. Nevertheless, the roles of m^6^A in CLL remain largely unexplored. Recent findings from our group suggest that CLL cells exhibit elevated expression of METTL3 protein, an RNA methyltransferase responsible for depositing m6A on mRNA. Correspondingly, there is also an upregulation of m6A on mRNA in CLL cells. Notably, a higher abundance of METTL3 protein correlates with the need for early treatment in CLL patients. Further investigations into the molecular mechanism of METTL3 in CLL have revealed its involvement in regulating the RNA splicing network, and its association with CLL aggressiveness. Subsequent analysis of FCR resistance will illustrate whether m^6^A modification and RNA splicing dysregulation may play a role in this aspect^[57]^.

### Clonal and subclonal evolution

Genetic lesions in CLL may change over time, leading to clonal evolution and the emergence of more aggressive clones or subclones. Clonal evolution is often accelerated in cells treated with chemotherapy that induces DNA damage, leading to the accumulation of genetic aberrations and increased genetic complexity, promoting progression and chemorefractoriness. Recent genomic studies have demonstrated significant intratumoral heterogeneity within CLL that is characterized by the presence of genetically diverse clones and subclones that interact and compete. Clones and subclones carrying genetic lesions that resulted from genotoxic chemotherapy, along with *TP53* aberrancies that prevent DNA repair, are resistant to and selected by CIT. As a result, clonal evolution occurs more frequently in patients with CLL receiving CIT, while clonal architecture remains stable in untreated CLL or in CLL treated by targeted therapy^[65,66]^.

## DISCUSSION AND CONCLUSIONS

FCR remains a viable option for long-term durable remission in patients with IGHV mutated disease without del(11q) or del(17p) who desire a short therapy course, after proper counseling of the not insignificant risk of therapy-related myeloid neoplasm (6.3%, 84% of which are fatal events). Recently, a 17-gene expression signature developed using a cohort of CLL patients at MD Anderson and validated in the CLL8 cohort allowed researchers to pinpoint patients with *IGHV*-unmutated CLL who were more likely to achieve durable remissions with FCR, although prospective validation is required^[49]^. While our paper describes known mechanisms of resistance against conventional chemoimmunotherapy, some of the resistance mechanisms may also apply to novel agents, including genomic instability (by facilitating the evolution of clones resistant to selective pressure of ibrutinib^[67]^), and high levels of pro-proliferative stimuli driven by *MYC* leading to clonal evolution and driving transformation to Richter’s syndrome. We expect advances in multi-omics to examine the complex interplay between somatic mutations and transcriptomic, epigenomic, and proteomic changes to identify factors underlying the heterogeneous evolution of CLL, thereby allowing a comprehensive framework to select patients for different treatments beyond genomic alterations. The main challenge for the future is translating multi-omics findings into personalized genetic-driven approaches to improve the clinical management of patients to allow long-term remissions with minimal toxicity. Despite these challenges, genomic changes associated with resistance to novel agents are increasingly being uncovered, and we hope further multi-omics studies will help guide genomic-based interventions in CLL.

## DECLARATIONS

### Copyright

© The Author(s) 2024.
